# Selective Serotonin Reuptake Inhibitors: Antimicrobial Activity Against ESKAPEE Bacteria and Mechanisms of Action

**DOI:** 10.3390/antibiotics14010051

**Published:** 2025-01-08

**Authors:** Thiago Hideo Endo, Mariana Homem de Mello Santos, Sara Scandorieiro, Bruna Carolina Gonçalves, Eliana Carolina Vespero, Márcia Regina Eches Perugini, Wander Rogério Pavanelli, Gerson Nakazato, Renata Katsuko Takayama Kobayashi

**Affiliations:** 1 Laboratory of Basic and Applied Bacteriology, Department of Microbiology, Center of Biological Sciences, Universidade Estadual de Londrina, Londrina 86057-970, Brazil; th.endo@uel.br (T.H.E.); mellomariana28@uel.br (M.H.d.M.S.); bruna.carolina@uel.br (B.C.G.); gnakazato@uel.br (G.N.); 2 Laboratory of Innovation and Cosmeceutical Technology, Department of Pharmaceutical Sciences, Center of Health Sciences, Hospital Universitário de Londrina, Londrina 86038-350, Brazil; sarascandorieiromicro@gmail.com; 3 Laboratory of Clinical Analysis Microbiology Sector, Department of Pathology, Clinical and Toxicological Analysis, Center of Health Sciences, Hospital Universitário de Londrina, Londrina 86038-350, Brazil; eliana.vespero@gmail.com (E.C.V.); marciaperugini@hotmail.com (M.R.E.P.); 4 Laboratory of Experimental Protozoology, Department of Pathological Sciences, Center of Biological Sciences, Universidade Estadual de Londrina, Londrina 86057-970, Brazil; wanderpavanelli@uel.br

**Keywords:** SSRI, drug repositioning, efflux pump inhibitors, oxidative stress, multidrug resistance

## Abstract

**Background:** Multidrug-resistant bacteria cause over 700,000 deaths annually, a figure projected to reach 10 million by 2050. Among these bacteria, the ESKAPEE group is notable for its multiple resistance mechanisms. Given the high costs of developing new antimicrobials and the rapid emergence of resistance, drug repositioning offers a promising alternative. **Results:** This study evaluates the antibacterial activity of sertraline and paroxetine. When tested against clinical and reference strains from the ESKAPEE group, sertraline exhibited minimum inhibitory concentration (MIC) values between 15 and 126 μg/mL, while the MIC values for paroxetine ranged from 60 to 250 μg/mL. Both drugs effectively eradicated bacterial populations within 2 to 24 h and caused morphological changes, such as protrusions and cellular fragmentation, as shown by electron scanning microscopy. Regarding their mechanisms of action as antibacterials, for the first time, increased membrane permeability was detected, as evidenced by heightened dye absorption, along with the increased presence of total proteins and dsDNA in the extracellular medium of *Escherichia coli* ATCC2 25922 and *Staphylococcus aureus* ATCC 25923, and oxidative stress was also detected in bacteria treated with sertraline and paroxetine, with reduced efficiency observed in the presence of antioxidants and higher levels of oxygen-reactive species evidenced by their reaction with 6-carboxy-2′,7′-dichlorodihydrofluorescein diacetate. The drugs also inhibited bacterial efflux pumps, increasing ethidium bromide accumulation and enhancing tetracycline activity in resistant strains. **Conclusions:** These findings indicate that sertraline and paroxetine could serve as alternative treatments against multidrug-resistant bacteria, as well as efflux pump inhibitors (EPIs), and they support further development of antimicrobial agents based on these compounds.

## 1. Introduction

Since Fleming’s discovery, a continuous race has emerged between the development of new antimicrobials and bacterial resistance. Microbial resistance can arise as a consequence of selective pressure imposed on microorganisms due to the excessive use of antimicrobials in human and veterinary medicine [1].

Multidrug-resistant (MDR) bacteria represent a global challenge, with their prevalence increasing over the past decade. They are responsible for over 700,000 deaths annually [2], and this number may reach 10 million by 2050, surpassing deaths caused by cancer, diabetes, and car accidents, resulting in costs that are expected to exceed $100 trillion [3]. This situation has become even more alarming after the COVID-19 pandemic, during which antimicrobial resistance increased [4]. Studies indicate that more than 70% of pathogenic bacteria exhibit resistance to at least one of the antimicrobials currently in use, with this percentage expected to rise annually [5].

Bacterial resistance can be developed (by random mutations) or obtained by mechanisms of horizontal gene transfer, such as transformation, transduction, and conjugation [6]. The molecular mechanisms of antibiotic resistance are grouped into five categories: active efflux (efflux pumps), changes in membrane permeability, inactivation of antibiotics by enzymes, changes in the antibiotic target, and duplication of the antibiotic target [7,8].

Among multidrug-resistant bacteria, those that are included in the ESKAPEE group (*Enterococcus faecium*, *Staphylococcus aureus*, *Klebsiella pneumoniae*, *Acinetobacter baumannii*, *Pseudomonas aeruginosa*, *Enterobacter* spp., and *Escherichia coli*) stand out, owing to their ability to quickly develop resistance to antimicrobials, their high association with healthcare-associated infections, and their consequent difficulty to treat, thus representing a great threat to public health [9,10]. The ESKAPEE group is characterized by a high incidence of multidrug-resistant strains and a high capability of horizontal gene transfer, facilitating the acquisition and dissemination of resistance genes [11,12]. It is estimated that five out of six leading pathogens associated with resistance-related deaths belong to the ESKAPEE group [13].

The acronym ESKAPE was coined after these pathogens were placed on a priority list published in 2017 by the World Health Organization, which described pathogens requiring the urgent development of novel antibiotics [9,14,15]. Later, some authors added *Escherichia coli* to the group; underscoring that it is a relevant member of the Enterobacteriaceae group and capable of producing enzymes involved in antibiotic resistance, such as extended-spectrum beta-lactamases (ESBLs) and carbapenemases, they classified this bacterium as a priority pathogen and formed the acronym ESKAPEE [16].

Due to the high hazard that the bacteria from the ESKAPEE group pose to public health, multiple strategies have been studied and applied for the control of these resistant and pathogenic microorganisms. These strategies include bacteriophage therapy, antimicrobial combinations, photodynamic therapy, antimicrobial peptides, and nanomaterials [17]. Another strategy that has shown efficiency in the control of ESKAPEE pathogens is the use of efflux pump inhibitors (EPIs) [10].

Given the growing concern over MDR bacteria, there is a need to develop new classes of antimicrobials. However, the high cost, estimated to be between 800 million and 1 billion dollars, combined with the rapid development of resistance, poses scientific and economic challenges to the pharmaceutical industry. This scenario has also led to modifications and combinations of existing antimicrobials, rather than the development of new antimicrobial classes [18,19].

In the absence of the development of novel antibiotics, drug repositioning has emerged as a strategy for treating multidrug-resistant bacteria. Drug repositioning is defined as “the discovery of new therapeutic opportunities for existing drugs” and brings a series of advantages, such as previous knowledge about the pharmacokinetics, pharmacodynamics, and toxicity of the drugs under investigation, since their safety has already been evaluated for their original application. This strategy reduces the duration of preclinical studies of the chosen drug, shortening its regulatory process from between 10 and 17 years to between only 3 and 12 years, thus providing great economical advantages [20]. The drug repositioning strategy has been previously studied for treating bacteria from the ESKAPEE group, with promising results [21].

Sertraline (SER) and paroxetine (PAR) are widely used for treating various degrees of depression, anxiety, and obsessive–compulsive disorder. Both belong to the class of selective serotonin reuptake inhibitors (SSRIs) [22]. Their mechanism of action involves targeting the serotonin transporter, responsible for the reuptake of 5-hydroxytryptamine (5-HT) in serotonergic neurons. Thus, the inhibition of 5-HT reuptake results in increased extracellular levels of 5-HT, generating an antidepressant activity [23,24]

Considering the known effects of SSRIs on microorganisms, but the limited understanding of their mechanisms of action [25], this study aims to characterize the mechanisms responsible for the antimicrobial activity of SER and PAR. Therefore, this study is the first to investigate how SER and PAR change bacterial membrane permeability and induce oxidative stress. Considering the slow and expensive development of new antibiotic classes, and the emergence of multidrug-resistant bacteria in public and animal health, this study explores the potential of repurposing SER and PAR as antimicrobials for treating ESKAPEE bacteria, while analyzing their mechanisms of antimicrobial action.

## 2. Results

### 2.1. Determination of Minimal Inhibitory Concentration (MIC) and Minimal Bactericidal Concentration (MBC)

The reference strains *E. coli* ATCC 25922 and *S. aureus* ATCC 25923, as well as multidrug-resistant strains from the ESKAPEE group, were tested in our study. The MIC values of SER ranged from 15 to 126 µg/mL, and its MBC values ranged from 30 to 500 µg/mL. As for PAR, its MIC and MBC values ranged from 60 to 255 µg/mL and 127.5 to 2040 µg/mL, respectively, as described in Table 1. Both SER and PAR were able to inhibit the growth of all tested bacteria.

A Wilcoxon test indicated that both the MIC and MBC of SER and PAR were statistically distinct (*p*-value = 0.0078 for MIC and *p*-value = 0.0039 for MBC, with a significance level of 0.05). It also indicated that the MICs and MBCs of PAR were higher than those of SER, as shown by the sum of positive ranks (36 for MIC and 45 for MBC). Furthermore, the Mann–Whitney test indicated that there were no significant differences in MIC and MBC between standard strains and clinical isolate from *E. coli* and *S. aureus*.

### 2.2. Time–Kill Assay

**Figure 1:**
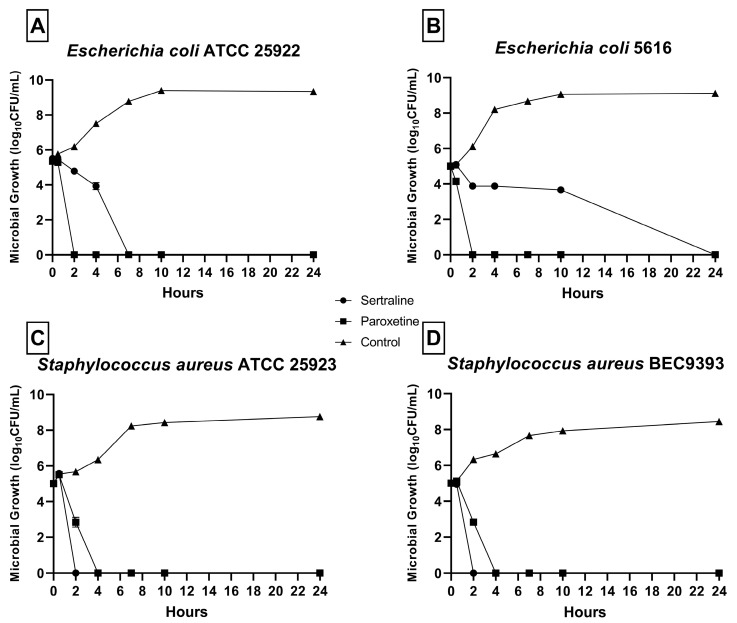
Time–kill curves of bacteria from the ESKAPEE group treated with sertraline (SER) and paroxetine (PAR) at minimal bactericidal concentration. (**A**) *Escherichia coli* ATCC 25922. (**B**) *Escherichia coli* 5616. (**C**) *Staphylococcus aureus* ATCC 25923. (**D**) *Staphylococcus aureus* BEC9393.

As shown in Figure 1, a reduction in CFU/mL was detected in all bacterial strains in the presence of SER and PAR. No microbial growth was detected after 24 h at any MBC concentration. For *E. coli* ATCC 25922 (Figure 1A), no growth was detected after 2 h of exposure to SER and 8 h of exposure to PAR. For *E. coli* 5616 (Figure 1B), no growth was detected after 24 h of exposure to SER and 2 h of exposure to PAR. Finally, for *S. aureus* ATCC 25923 (Figure 1C) and *S. aureus* BEC9393 (Figure 1D), growth was inhibited after 2 h of exposure to SER and 4 h of exposure to PAR.

### 2.3. Scanning Electron Microscopy

**Figure 2:**
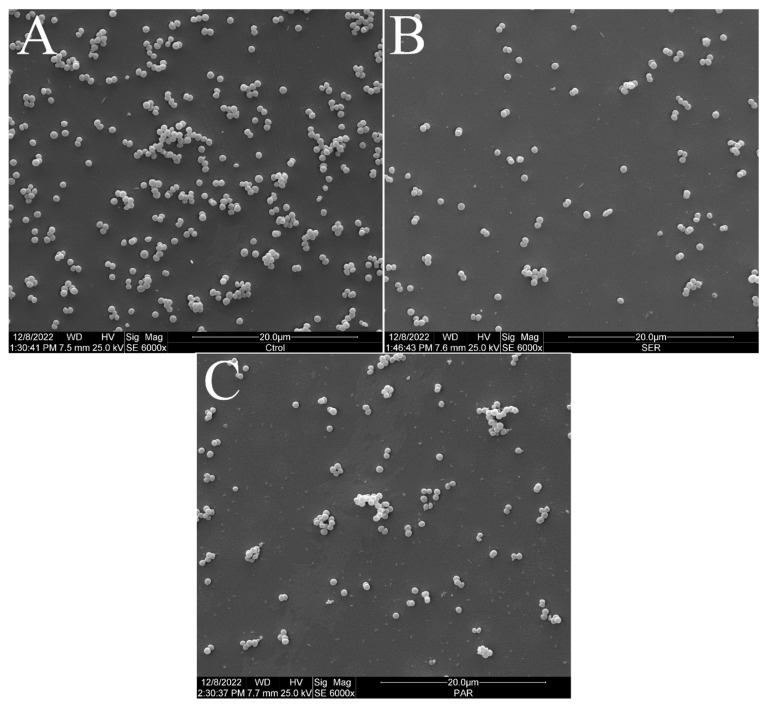
Scanning electron micrographs of *S. aureus* ATCC 25923 treated with sertraline and paroxetine, at 6000× magnification. (**A**) Untreated control. (**B**) Sertraline-treated cells. (**C**) Paroxetine- treated cells.

Figure 2 shows the scanning electron micrographs of *S. aureus* ATCC 25923 at 6000× magnification. The untreated control group exhibited the typical morphology, size, and arrangement of *S. aureus* (Figure 2A). However, the groups treated with SER (Figure 2B) and PAR (Figure 2C) showed a reduced bacterial population compared to the untreated control group.

**Figure 3:**
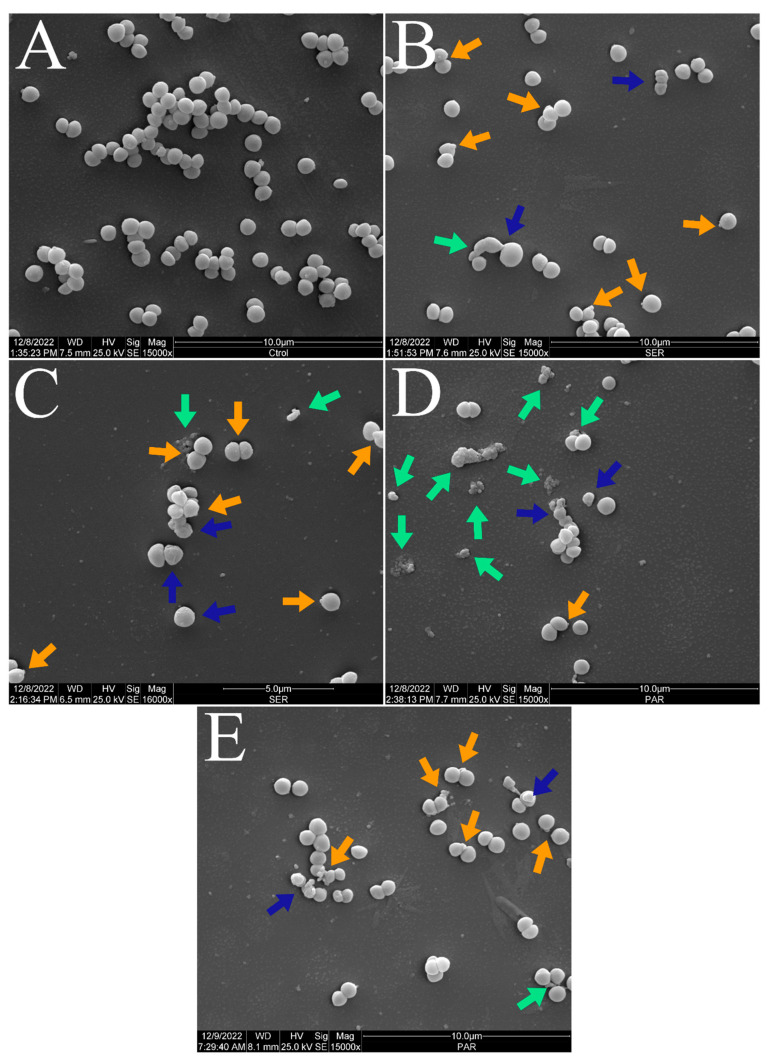
Scanning electron micrographs of *S. aureus* ATCC 25923 treated with sertraline and paroxetine, at 15,000× magnification. (**A**) Untreated control. (**B**,**C**) Sertraline-treated cells. (**D**,**E**) Paroxetine-treated cells. Morphological alterations (blue arrows), cellular protrusions (orange arrows), and formation of cellular debris (green arrows).

**Figure 4:**
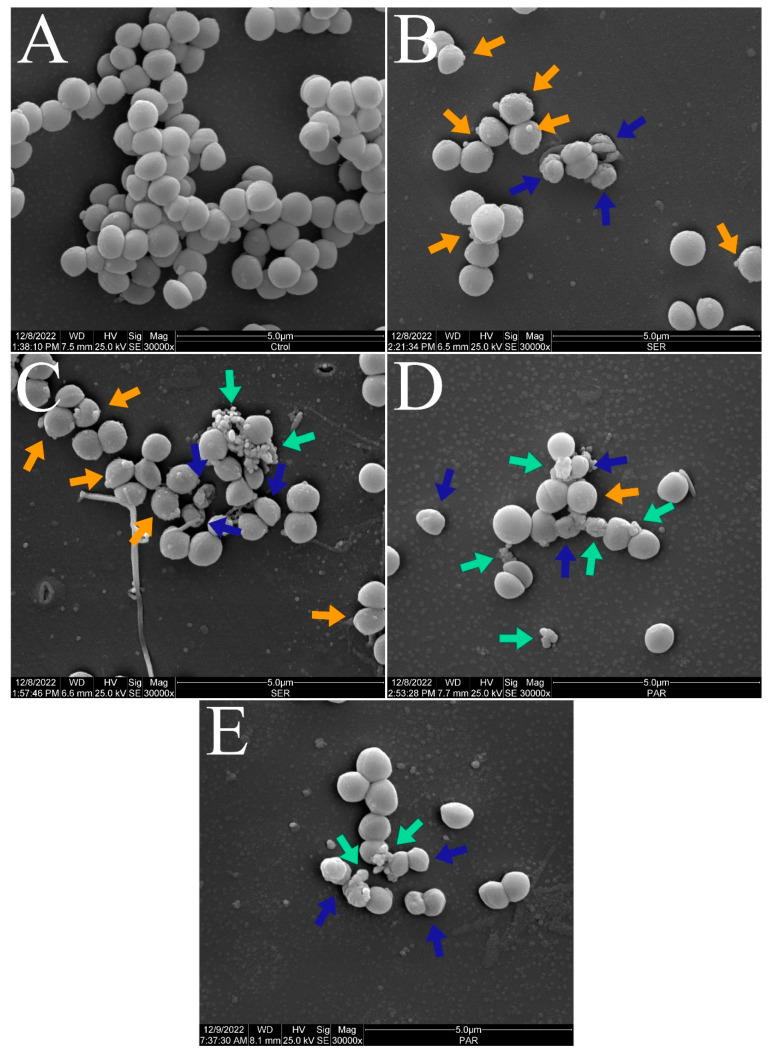
Scanning electron micrographs of *S. aureus* ATCC 25923 treated with sertraline and paroxetine, at 30,000× magnification. (**A**) Untreated control. (**B**,**C**) Sertraline-treated cells. (**D**,**E**) Paroxetine-treated cells. Morphological alterations (blue arrows), cellular protrusions (orange arrows), and formation of cellular debris (green arrows).

Figure 3 and Figure 4 show the scanning electron micrographs of *S. aureus* ATCC 25923 at 15,000× and 30,000× magnifications. The untreated control group showed the typical morphology, size, and arrangement of *S. aureus* (Figure 3A and Figure 4A). Nevertheless, groups treated with SER (Figure 3B,C and Figure 4B,C) and PAR (Figure 3D,E and Figure 4D,E) displayed morphological alterations (blue arrows), cellular protrusions (orange arrows), and formation of cellular debris (green arrows). Treatment with SER seemed to induce slightly more apparent cellular protrusions, whereas bacterial cells treated with PAR presented more cellular debris.

### 2.4. Determination of Subinhibitory Antibacterial Concentrations for Mechanism Assays

To select the most suitable treatment time and concentration of SER and PAR, a 4 h time–kill assay was performed. This assay aimed to identify the conditions that resulted in no greater than 1-log reduction in bacterial viability over the longest possible treatment duration. After a 4 h treatment with SER and PAR at MIC and ½ MIC concentrations, viable cells of *E. coli* ATCC 25922 and *S. aureus* ATCC 25923 were detected. Notably, PAR- treated cells showed a reduction in cell concentration superior to 1 log only at MIC and ½ MIC concentrations after 4 h, as detailed in Table 2. Thus, the conditions for SER and PAR were selected as ½ MIC for 3 h, as these presented better cell viability.

### 2.5. Membrane Permeability Evaluation

#### 2.5.1. Measurement of Permeability with High-Molecular-Weight Dyes

**Figure 5:**
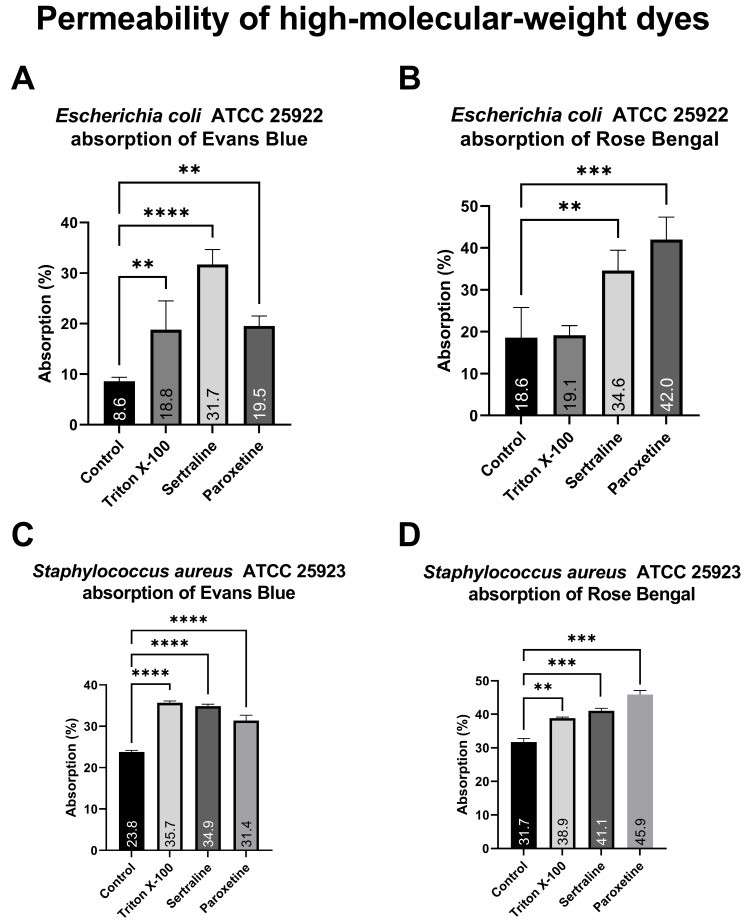
Absorption of Evans blue and Rose Bengal by *Escherichia coli* ATCC 25922 and *Staphylococcus aureus* ATCC 25923 treated with sertraline and paroxetine. (**A**) *Escherichia coli* ATCC 25922: absorption of Evans blue. (**B**) *Escherichia coli* ATCC 25922: absorption of Rose Bengal. (**C**) *Staphylococcus aureus* ATCC 25923: absorption of Evans blue. (**D**) *Staphylococcus aureus* ATCC 25923: absorption of Rose Bengal. (**) *p*-value ≤ 0.01, (***) *p*-value ≤ 0.001, (****) *p*-value ≤ 0.0001.

As illustrated in Figure 5, bacterial cells of the different *E. coli* treatment groups exposed to Evans blue (A) absorbed between 9.5% and 31.7% of the dye, whereas bacteria exposed to Rose Bengal (B) absorbed between 18.6% and 42.0%. Similarly, the different *S. aureus* treatment groups exposed to Evans blue (C) presented dye absorption ranging from 8.77% to 35.67%, whereas the absorption of those exposed to Rose Bengal (D) ranged from 22.87% to 45.89%. Both treatments significantly altered the absorption of both dyes, except for Triton X-100 on *E. coli*. The increased absorption of high-molecular-weight dyes demonstrates a change in the membrane permeability of the tested bacteria, which is reinforced by the positive control (Triton X-100).

#### 2.5.2. Quantification of Biomolecule Leakage

**Figure 6:**
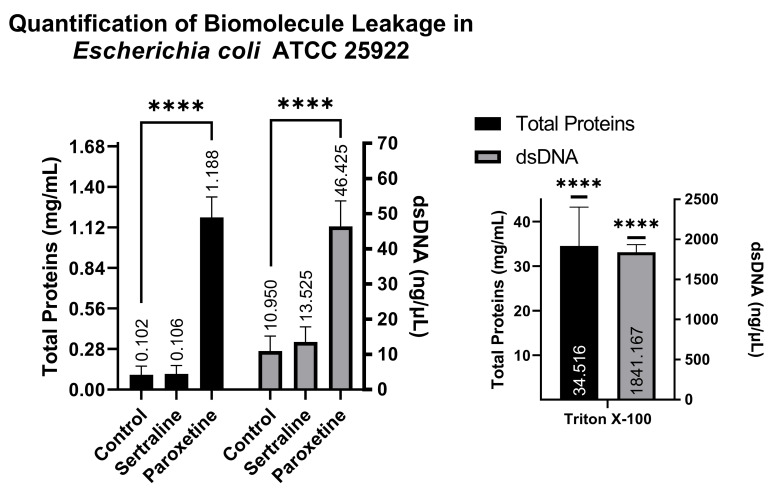
Quantification of total proteins and double-stranded DNA from the supernatants of *Escherichia coli* ATCC 25922 treated with sertraline and paroxetine. (****) *p*-value ≤ 0.0001.

A comparison of NanoDrop readings allows for the verification of whether treatments with SER and PAR cause leakage of biomolecules from the intracellular to the extracellular environment as a result of altered membrane permeability. The NanoDrop readings indicated that the *E. coli* group treated with SER showed 0.106 mg/mL of total proteins and 13.52 ng/μL of dsDNA; the group treated with PAR showed 1.188 mg/mL of total proteins and 46.42 ng/μL of dsDNA; the untreated control group showed 0.102 mg/mL of total proteins and 10.95 ng/μL of dsDNA; and finally, the control group treated with Triton X-100 exhibited 34.516 mg/mL of total proteins and 1841.167 ng/μL of dsDNA. These findings suggest that PAR treatment can induce biomolecule leakage in *E. coli*, as observed in Figure 6, which is reinforced by the positive control (Triton X-100).

**Figure 7:**
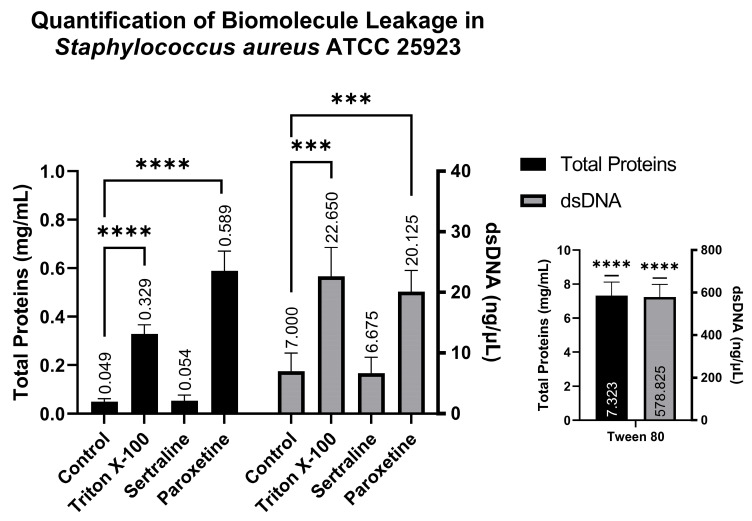
Quantification of total proteins and double-stranded DNA from the supernatants of *Staphylococcus aureus* ATCC 25923 treated with sertraline and paroxetine. (***) *p*-value ≤ 0.001, (****) *p*-value ≤ 0.0001.

Similarly to the quantification of biomolecules in *E. coli*, analysis conducted using the supernatants of *S. aureus* revealed the following: the SER-treated group showed 0.054 mg/mL of total proteins and 6.67 ng/µL of dsDNA; the PAR-treated group showed 0.589 mg/mL of total proteins and 20.12 ng/µL of dsDNA; the Tween 80-treated group showed 7.323 mg/mL of total proteins and 578.82 ng/µL of dsDNA; and finally, the Triton X-100-treated group showed 0.329 mg/mL of total proteins and 22.65 ng/µL of dsDNA. These results also indicate that PAR can induce biomolecule leakage, as observed in Figure 7, which is reinforced by the positive controls (Triton X-100 and Tween 80).

### 2.6. Evaluation of Efflux Pump Inhibition

#### 2.6.1. Resistance Modulation Assay

As shown in Table 3, *E. coli* XL1-Blue exhibited a mean MIC of 312.5 µg/mL when treated with tetracycline (TET) alone, 130.12 µg/mL when treated with TET and SER, and 11.88 µg/mL when treated with TET and PAR, at subinhibitory concentrations in all these cases. Conversely, *E. coli* K12 presented an average MIC of 0.255 µg/mL when treated with TET alone, 0.15 µg/mL when treated with TET and SER, and 0.3 µg/mL when treated with TET and PAR, also at subinhibitory concentrations.

The results demonstrate that SER and PAR were able to significantly modulate tetracycline resistance in *E. coli* XL1-Blue, a resistance mechanism attributed exclusively to efflux pumps. This finding is further supported by the lack of significant modulation observed in the K12 strain, which shares a high level of genomic homology with the XL1-Blue strain, but lacks tetracycline resistance.

#### 2.6.2. Ethidium Bromide Accumulation Assay

**Figure 8:**
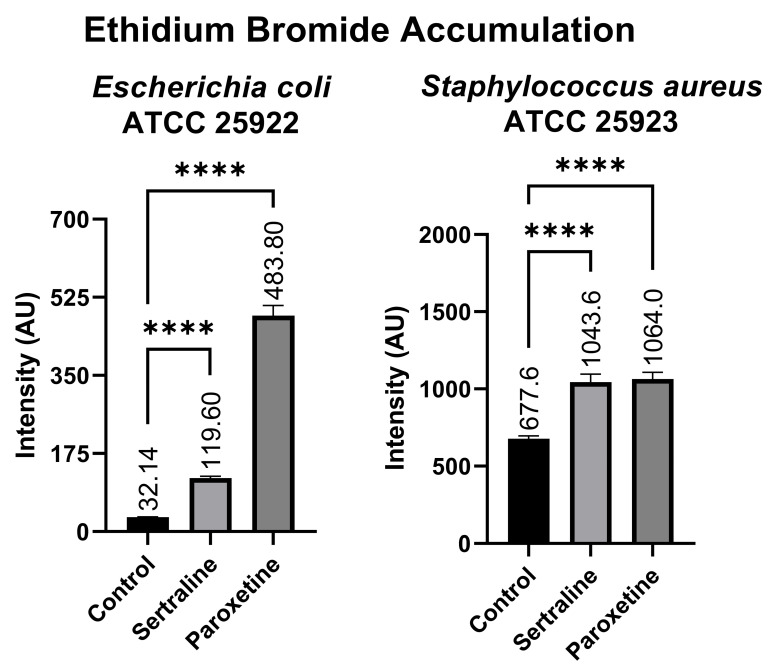
Ethidium bromide accumulation in suspensions of *Escherichia coli* ATCC 25922 and *Staphylococcus aureus* ATCC 25923 treated with sertraline and paroxetine. (****) *p*-value ≤ 0.0001.

Figure 8 shows that the *E. coli* bacterial suspensions displayed a mean fluorescence of 119.60 AU when treated with SER, 483.80 AU when treated with PAR, and 32.14 AU in the untreated control group. In contrast, the bacterial suspensions of *S. aureus* exhibited 1043.60 AU of fluorescence when treated with SER, 1064.00 AU when treated with PAR, and 677.60 AU in the untreated control group. Compared to the control group, both treatments resulted in a statistically significant increase in ethidium bromide accumulation in *E. coli* and *S. aureus*, denoting the inhibition of efflux pumps, which are responsible for the expulsion of ethidium bromide from the intracellular environment.

### 2.7. Oxidative Stress Evaluation

#### 2.7.1. Detection of the Effect of Antioxidants on the Antibacterial Activity of SER and PAR

When treated with SER alone, *E. coli* and *S. aureus* exhibited mean MIC values of 25.5 µg/mL and 27.0 µg/mL (Table 4), respectively. The combination of SER with glutathione at subinhibitory concentrations inhibited bacterial growth at 13.7 µg/mL for *E. coli* and 62.5 µg/mL for *S. aureus*. Similarly, combining SER with ascorbic acid at a subinhibitory concentration resulted in a mean MIC of 15.6 µg/mL for *E. coli* and 62.5 µg/mL for *S. aureus*.

Both bacteria, when tested with PAR alone, exhibited a mean MIC of 127.5 µg/mL. However, the treatment with SER plus glutathione at a subinhibitory concentration resulted in a mean MIC of 209.2 µg/mL for *E. coli* and 250.0 µg/mL for *S. aureus*. Treatment with PAR in combination with ascorbic acid at a subinhibitory concentration presented a mean MIC of 416.7 µg/mL for *E. coli* and 0.255 µg/mL for *S. aureus*.

The positive control, AgNO_3_, showed a mean MIC of 62.5 µM for *E. coli* and 93.5 µM for *S. aureus*. When combined with glutathione at subinhibitory concentrations, AgNO_3_ presented an MIC exceeding 1 mM for both tested bacteria. AgNO_3_, in combination with ascorbic acid, exhibited a mean MIC of 127.5 µM for *E. coli*.

The other positive control, H_2_O_2_, showed a mean MIC of 0.7 mM for *E. coli* and 2.0 mM for *S. aureus*. In combination with glutathione at subinhibitory concentrations, H_2_O_2_ showed a mean MIC of 1.5 mM for *E. coli* and 32.0 mM for *S. aureus*. Finally, the negative control, ampicillin, exhibited a mean MIC of 0.25 µg/mL, alone and in combination with glutathione at subinhibitory concentrations.

These results suggest that antioxidants influence the antibacterial activity of SER against *S. aureus* and PAR against both bacteria, indicating that these treatments can induce oxidative stress. These findings are reinforced by the positive and negative controls employed in the experiment.

#### 2.7.2. Measurement of Reactive Oxygen Species

**Figure 9:**
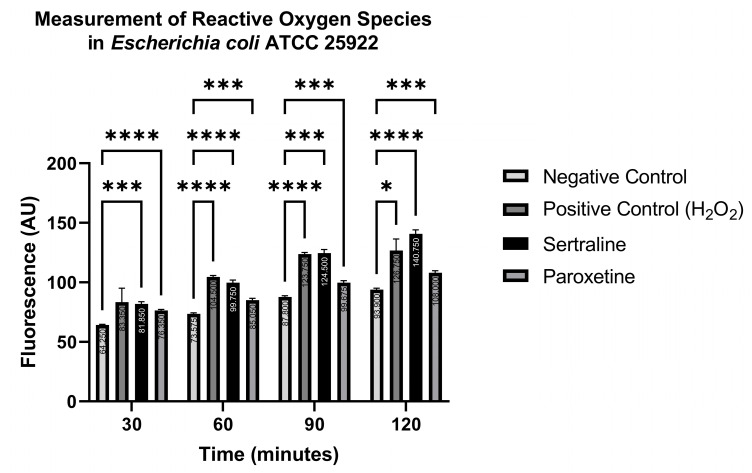
Measurement of reactive oxygen species in *Escherichia coli* ATCC 25922 treated with sertraline and paroxetine, utilizing the fluorescent indicator 6-carboxy-2′,7′-dichlorodihydrofluorescein diacetate. (*) *p*-value ≤ 0.05, (***) *p*-value ≤ 0.001, (****) *p*-value ≤ 0.0001.

The indicator 6-carboxy-2′,7′-dichlorodihydrofluorescein diacetate (H_2_DCFDA) reacts with reactive oxygen species (ROS), converting the non-fluorescent indicator to a green-fluorescent form that is read at an emission wavelength of 500–550 nm and an excitation wavelength of 475 nm. Readings were carried out at 30, 60, 90, and 120 min. Figure 9 shows the ROS production in *E. coli* ATCC 25922. Significantly higher fluorescence levels were detected in the treatments with SER, PAR, and the positive control (H_2_O_2_), compared to the negative control (PBS), at all time points, except for the positive control at 30 min. Even though higher fluorescence was detected in the positive control at 30 min compared to the negative control, this difference was not statistically significant. These results indicate that both SER and PAR are capable of inducing the formation of ROS in *E. coli* ATCC 25922.

**Figure 10:**
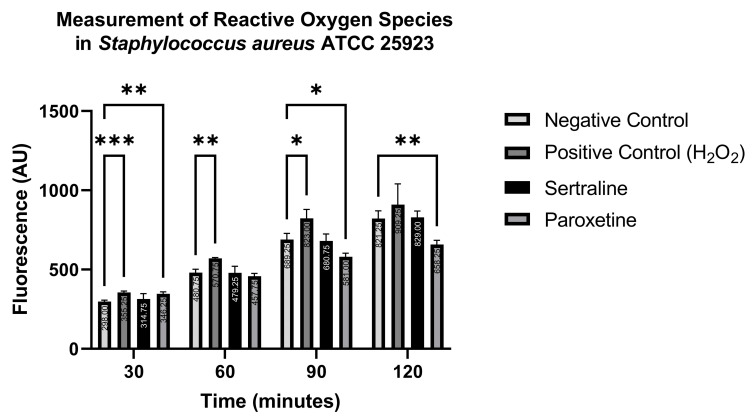
Measurement of reactive oxygen species in *Staphylococcus aureus* ATCC 25923 treated with sertraline and paroxetine, utilizing the fluorescent indicator 6-carboxy-2′,7′-dichlorodihydrofluorescein diacetate. (*) *p*-value ≤ 0.05, (**) *p*-value ≤ 0.01, (***) *p*-value ≤ 0.001.

In contrast, Figure 10 shows that ROS production in *S. aureus* ATCC 25923 was statistically higher than the negative control when treated with the positive control only at 30, 60, and 90 min, and with paroxetine at 30 min. Conversely, paroxetine treatment at 90 and 120 min resulted in significantly lower ROS production compared to the negative control. These results suggest that SER does not induce ROS formation in *S. aureus* ATCC 25923. Moreover, PAR initially stimulates ROS production, but this effect is reduced after 60 min of treatment.

### 2.8. Resistance Inducement Assay

**Figure 11:**
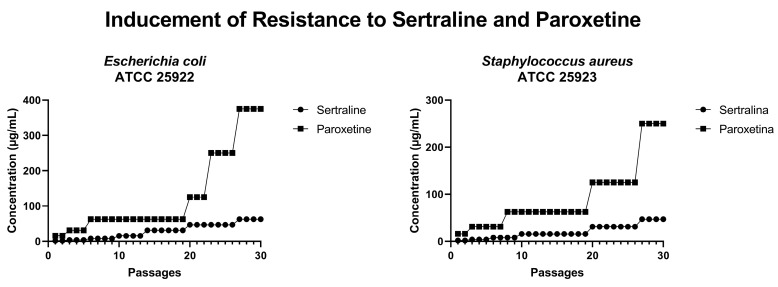
Concentrations of sertraline and paroxetine during resistance inducement in *Escherichia coli* ATCC 25922 and *Staphylococcus aureus* ATCC 25923.

In an attempt to induce bacterial resistance to SER and PAR, cultures of *E. coli* ATCC 25922 and *S. aureus* ATCC 25923 were subjected to 30 serial passages with progressively increasing concentrations of these drugs. As illustrated in Figure 11, after 30 passages, *E. coli* could grow in the presence of 62.4 µg/mL of SER (four times the MIC of SER) and 375.0 µg/mL of PAR (three times the MIC of PAR). Furthermore, *S. aureus* was able to grow in the presence of 46 µg/mL of SER (three times the MIC of SER) and 250 µg/mL of PAR (two times the MIC of PAR). Therefore, it is possible to conclude that neither *E. coli* nor *S. aureus* developed resistance to the tested drugs within 30 serial passages.

## 3. Discussion

Our study evaluated the antibacterial activity of SER and PAR against reference strains and MDR strains of the ESKAPEE group. It also sought to elucidate the mechanisms of action underlying this antibacterial activity. SER and PAR exhibited good antimicrobial activity against both the reference and MDR strains, displaying effects on bacterial morphology, membrane permeability, efflux pumps, and oxidative stress induction. To the best of our knowledge, this is one of the first studies to reveal the potential antibacterial mechanisms of action of SER and PAR.

The drugs inhibited the growth of and killed reference and MDR bacterial strains, including Gram-positive and Gram-negative bacteria. SER exhibited inhibitory activity at concentrations ranging from 15 to 126 μg/mL, while PAR showed inhibitory activity between 60 and 250 μg/mL. These findings align with the study of Muñoz-Bellido et al. [25], which reported the antibacterial activity of sertraline against *E. coli*, *K. pneumoniae*, *E. cloacae*, *A. baumannii*, and *S. aureus* at similar concentrations to those observed in this study. Likewise, Kruszewska et al. [26] identified antimicrobial activity of paroxetine against *S. aureus*, *E. coli*, and *P. aeruginosa*, although with different MIC values, likely due to methodological variations.

Among the tested bacteria, *P. aeruginosa* exhibited the highest MIC for SER (127.5 µg/mL), greater than the MICs observed in the other strains (15–60 µg/mL). Regarding the MBC values for SER, both *P. aeruginosa* and *E. cloacae* stood out with elevated MBCs (500 µg/mL), compared to the lower range observed in other strains (30–60 µg/mL). For the PAR MICs, *E. faecium* was notable for having the lowest value (60 µg/mL) compared to the higher MICs of the other strains (127.5–255 µg/mL). When analyzing the PAR MBCs, *P. aeruginosa*, *E. cloacae*, and *K. pneumoniae* had the highest values (2040 µg/mL, 2040 µg/mL, and 1020 µg/mL, respectively). Additionally, *E. faecium* was distinguished by its lower MBC (127.5 µg/mL) compared to the other strains (255–500 µg/mL). In summary, *P. aeruginosa* emerged as the most resistant strain to both SER and PAR, followed by *E. cloacae*, whereas *E. faecium* proved to be the most susceptible.

In the studies of Geronikaki et al. [27], the MIC values for streptomycin were 0.13 µg/mL for *S. aureus*, 0.03 µg/mL for *E. cloacae*, 0.1 µg/mL for *P. aeruginosa*, and 0.13 µg/mL for *E. coli*. The MBC values were 0.27 µg/mL for *S. aureus*, 0.07 µg/mL for *E. cloacae*, 0.2 µg/mL for *P. aeruginosa*, and 0.23 µg/mL for *E. coli*. For ampicillin, the MIC values were 0.17 µg/mL for *S. aureus*, 0.13 µg/mL for *E. cloacae*, 0.25 µg/mL for *P. aeruginosa*, and 0.18 µg/mL for *E. coli*. The MBC values were 0.18 µg/mL for *S. aureus*, 0.2 µg/mL for *E. cloacae*, 0.67 µg/mL for *P. aeruginosa*, and 0.27 µg/mL for *E. coli*. Compared to the data from this study, streptomycin and ampicillin generally exhibit lower MIC and MBC values, reflecting their established roles in targeting the tested bacterial species. In contrast, SER and PAR show higher MIC and MBC values (MIC: 15–127.5 µg/mL; MBC: 30–500 µg/mL), particularly against strains like *Pseudomonas aeruginosa* and *Enterobacter cloacae*. This suggests that while non-traditional compounds may act differently from antibiotics, they could offer alternative strategies for addressing bacterial infections, especially in innovative or combination therapies.

Likewise, the studies conducted by Rodrigues et al. [28] were performed with clinical isolates, where for *S. aureus*, the antimicrobials gentamicin, chloramphenicol, oxacillin, and meropenem showed MICs of 12.5, 6.25, 6.25, and 6.25 µg/mL, respectively, and MBCs of 100, 12.5, 6.25, and 6.25 µg/mL. For *P. aeruginosa*, the MICs of gentamicin, chloramphenicol, ceftazidime, and meropenem were 12.5, 6.25, 25, and 12.5 µg/mL, respectively, and the MBCs were 25, 25, 100, and 12.5 µg/mL. Compared to these data, the MIC and MBC concentrations of SER for *S. aureus* were slightly higher (MIC 15 µg/mL; MBC 30 µg/mL) than those found for gentamicin, chloramphenicol, oxacillin, and meropenem, while for *P. aeruginosa*, the MIC and MBC values were higher (127.5 µg/mL and 500 µg/mL, respectively. PAR showed even higher values (MIC 127.5–255 µg/mL and MBC 255–500 µg/mL) than the conventional antimicrobials. Although PAR presented higher MICs and MBCs, the MICs and MBCs of PAR for every tested bacterium were lower than the usually used therapeutic doses.

SER and PAR predominantly exhibited MBC values that were higher than their MIC values, indicating bacteriostatic activity at MIC concentrations and bactericidal activity at higher concentrations. These results aligns with those of Samanta et al. [29], who observed a bacteriostatic effect of SER on *Bacillus subtilis* UC564 and *Shigella dysenteriae* NCTC 599/52. The results from the subinhibitory concentration assays further support these findings. Specifically, *E. coli* and *S. aureus* were able to survive for up to 3 h in contact with SER and PAR at MIC concentrations, without a reduction in population exceeding 1 log. However, in the time–kill curve assays, *S. aureus* was eliminated within 2 h of exposure to SER, whereas *E. coli* was killed within 2 h of exposure to PAR at MBC concentrations.

Our study and that of Muñoz-Bellido et al. [25] show that the MIC and MBC values of SER and PAR for Gram-negative bacteria were higher than those for Gram-positive bacteria, suggesting a relatively positive activity of these drugs against Gram-positive bacteria. Additionally, the time–kill curve assay revealed that Gram-positive bacteria tend to be eliminated faster by SER (2 h) than by PAR (4 h), while Gram-negative bacteria are eliminated more rapidly by PAR (2 h) compared to SER (7 to 24 h).

Evans blue and Rose Bengal dyes possess high molecular weights. Therefore, they are more easily absorbed when alterations in the bacterial membrane occur. In the absorption assay using high-molecular-weight dyes, bacteria treated with SER and PAR exhibited increased absorption of Evans blue and Rose Bengal, indicating that these drugs alter the permeability of treated cells. Similar findings were reported by Aguilar-Toalá et al. [30], who assessed changes in bacterial membrane permeability caused by chia seed-derived peptides using analogous methodologies, and detected an increased absorption of dye by *E. coli*. Similarly, Nogueira and collaborators [31] demonstrated the ability of certain terpenes and phenylpropanoids to increase crystal violet uptake in *E. coli* and decrease crystal violet uptake in *S. aureus*, with both scenarios indicating alterations in bacterial membrane permeability. The results of this study are further supported by the positive control—the detergent triton X-100, which is known to increase cellular permeability [32].

The quantification of biomolecule leakage, using a Nanodrop spectrophotometer, revealed the presence of total proteins and dsDNA in the extracellular medium, indicating that the bacterial membrane suffered damage, resulting in the leakage of intracellular biomolecules into the extracellular environment [33]. This biomolecule leakage methodology has already been used to identify permeability alterations in *E. coli* and *S. aureus* in other studies [34,35], with an increase in dsDNA and total proteins also detected in bacteria treated with Salicylic Acid Microcapsules and certain antihistamines. Thus, the results from the high-molecular-weight dye absorption assay, combined with those from the quantification of total proteins and dsDNA, demonstrate that bacterial cells treated with PAR experience altered permeability and likely sustained membrane damage. This conclusion is supported by the significantly higher amounts of dsDNA (46.43 ng/µL for *E. coli* and 20.13 ng/µL for *S. aureus*) and total proteins (11.19 mg/mL for *E. coli* and 10.59 mg/mL for *S. aureus*) found in the extracellular material of the PAR-treated group compared to the untreated group (10.95 ng/µL of dsDNA and 0.10 mg/mL of total proteins for *E. coli*; 7.00 ng/µL of dsDNA and 0.05 mg/mL of total proteins for *S. aureus*). In contrast, SER-treated bacteria did not exhibit significant biomolecule leakage, indicating that SER alters bacterial membrane permeability, but it does not cause membrane damage.

The evaluation of efflux pump inhibition suggests that both drugs, SER and PAR, are capable of inhibiting efflux pumps. In the tetracycline-resistant *E. coli* XL1-Blue, treatment with tetracycline alone resulted in a significantly higher MIC (312.50 µg/mL) compared to tetracycline combined with SER (113.87 µg/mL) or PAR (11.88 µg/mL) at subinhibitory concentrations. Ayaz et al. [36] also observed improved activity of some antimicrobials when combined with SER. Tests conducted on the tetracycline-sensitive bacteria *E. coli* ATCC K12 reaffirmed that the aforementioned results were due to efflux pump inhibition, as there were no significant differences between the MIC of the group treated with tetracycline alone (0.26 µg/mL) and those of the groups treated with tetracycline and SER at subinhibitory concentrations (0.13 µg/mL) or tetracycline and PAR at subinhibitory concentrations (0.30 µg/mL). Even though both of the strains used in this study have high genetic homology, one remarkable difference between them is the presence of efflux pumps that confer resistance to tetracycline.

Also indicating efflux pump inhibition by SER and PAR, the ethidium bromide accumulation tests showed greater ethidium bromide accumulation in the tested bacteria treated with SER and PAR. For instance, *E. coli* showed a statistically significant increase in ethidium bromide accumulation when treated with SER (119.6 a.u.) and PAR (483.8 a.u.), compared to the untreated control (32.14 a.u.). Similarly, *S. aureus* exhibited a statistically significant increase in ethidium bromide accumulation in cells treated with SER (1043.6 a.u.) and PAR (1064.0 a.u.), compared to the untreated control (677.6 a.u.). Mahey et al. [37] evaluated efflux pump inhibition induced in *S. aureus* by antifungal azoles using the same methodology. Other studies in the literature [38] indicate that SER can act as a substrate for the *E. coli* AcrAB efflux pump, leading to the accumulation of Nile red and ethidium bromide dyes. Additionally, Kaatz et al. [39] demonstrated that a structural variant of PAR increased the accumulation of acriflavine, pyronin Y, and ethidium bromide in *S. aureus*. In summary, the literature not only indicates that the ethidium bromide accumulation methodology is efficient for evaluating efflux pump inhibition in *E. coli* and *S. aureus*, but also, as shown in the present study, it points out that SER and PAR exhibit the ability to inhibit efflux pumps.

The drugs SER and PAR also demonstrated the ability to induce oxidative stress in bacterial cells. Studies by Liao et al. [40] have highlighted the effects of antioxidants on an antimicrobial causing oxidative stress, using a methodology similar to that employed in this study. In the assay evaluating the impact of antioxidants on antimicrobial activity, it was shown that PAR could induce oxidative stress in *E. coli*, as the bacteria treated with PAR alone exhibited a lower MIC (127.5 µg/mL) than those treated with PAR in the presence of glutathione (209.2 μg/mL) or ascorbic acid (416.7 μg/mL). This effect was not observed in *E. coli* treated with SER, as bacteria treated with SER alone (25.5 μg/mL) had a higher MIC than those treated with SER in the presence of glutathione (13.7 μg/mL) or ascorbic acid (15.6 μg/mL).

For *S. aureus, however,* SER and PAR showed the potential to cause oxidative stress, as their MICs alone (27.0 μg/mL and 127.5 μg/mL, respectively) were lower than the MICs of bacteria treated with SER and PAR in the presence of glutathione and ascorbic acid (62.5 μg/mL and 250.0 μg/mL, respectively). The controls used in this test support its validity: the positive controls, AgNO_3_ and H_2_O_2_, which are known to cause oxidative stress, displayed lower MICs when used alone compared to when used in combination with glutathione or ascorbic acid at subinhibitory concentrations. Conversely, ampicillin served as a negative control, as its antimicrobial activity does not rely on oxidative stress. As expected, AMP showed little to no difference in MIC when used alone or in combination with glutathione at subinhibitory concentrations.

The measurements of ROS highlight the ability of SER and PAR to induce oxidative stress in *E. coli*, as both treatments were able to generate a statistically higher amount of ROS compared to the negative control at all tested time points. For *S. aureus, however,* the only treatment that showed a statistically significant increase was PAR at the 30 min time point. Other studies have reported on the quantification of ROS generated in bacteria using similar methodologies [41,42,43]. According to Scandorieiro et al. [44], an excessive increase in ROS leads to lipid peroxidation, which can reduce bacterial membrane fluidity, causing structural damage and potentially destroying membrane-associated proteins, including efflux pumps. Thus, oxidative stress may be the direct or indirect cause of changes in permeability, bacterial cell membrane damage, and efflux pump inhibition.

The scanning electron microscopy analysis (Figure 2, Figure 3 and Figure 4) showed morphologic alterations, such as cellular protrusions and the formation of cellular debris, in *S. aureus* ATCC 25923 treated with SER and PAR. However, treatment with SER seemed to induce more cellular protrusions, while treatment with PAR generated more cellular debris. This is likely due to the differences in the mechanisms of action of SER and PAR, with SER showing a lower ability to induce leakage of biomolecules, thus altering membrane permeability without causing significant damage to the membrane, and showing a greater ability to inhibit efflux pumps. In contrast, PAR exhibited greater biomolecule leakage, likely causing damage to the membrane. The scanning electron microscopy analysis results are supported by the results of the quantification of biomolecule leakage and the evaluation of oxidative stress, as the reduced fluidity of the bacterial membrane caused by lipidic peroxidation may result in morphological changes, consequently causing physical damage and leakage of biomolecules [45].

The induction of resistance to SER and PAR was not successful in *E. coli* and *S. aureus*, even after 30 passages. This is likely due to the multifactorial nature of the mechanisms of action of SER and PAR, which include changes in permeability, efflux pump inhibition, and the induction of oxidative stress.

Studies reported in the literature indicate that therapeutic doses of SER range from 25 to 100 mg, resulting in maximum drug concentrations between 25 and 55 ng/mL. Meanwhile, therapeutic dosages of PAR range between 12.5 and 50 mg, which can reach maximum concentrations exceeding 30 ng/mL [46]. Additionally, the literature also describes that SER overdoses can occur with the consumption of 500 to 6000 mg, and PAR overdoses can occur with the consumption of 800 to 2000 mg [47]. Although the comparison of MIC and MBC with therapeutic doses and overdoses is crude, the MIC and MBC concentrations of SER and PAR observed in this study are within the therapeutic range of these drugs, and remain below their overdose thresholds; however, the maximum concentration obtained with therapeutic doses is lower than the MICs and MBCs found in this study. Moreover, additional studies, particularly in vivo studies, are certainly necessary to confirm the safety and effectiveness of SER and PAR as antimicrobial agents. Furthermore, exploring strategies such as the use of SER and PAR as topical antimicrobials, or the employment of these drugs as potentiators of conventional antimicrobials (since these drugs inhibit efflux pumps), can further demonstrate that SER and PAR could be utilized in the fight against MDR bacteria. 

Future studies on the same topic as the present study should include in vivo trials to evaluate the effectiveness and safety of using SER and PAR as antimicrobial agents; more in-depth studies on the mechanisms of action of SER and PAR, especially concerning efflux pumps; studies on the combination of SER and PAR with conventional and alternative antimicrobials; and even the modification of these compounds to potentially create a new class of antibiotics. This study is one of the first to characterize the different mechanisms of action for SER and PAR, including oxidative stress induction, alterations in membrane permeability, and their capability to act as EPIs. Furthermore, it demonstrates their action against multidrug-resistant bacteria, which makes them a promising treatment alternative. However, more studies are needed concerning their applications in combating multidrug resistance in clinical, veterinary, or cosmetic settings.

## 4. Materials and Methods

### 4.1. Bacterial Strains

In this study, two American Type Culture Collection strains were used—*Escherichia coli* ATCC 25922 and *Staphylococcus aureus* ATCC 25923. Moreover, the standard strains *Escherichia coli* XL1-Blue and *Escherichia coli* K12 were also used.

The following multidrug-resistant bacterial isolates from the ESKAPEE group were also tested [48]:

- *E. faecium* 99455: resistant to streptomycin, ampicillin, teicoplanin, and levofloxacin. Isolated from urine at the University Hospital of Londrina (HU), Paraná State, Brazil.
- *S. aureus* BEC9393: resistant to cefoxitin, tetracycline, linezolid, chloramphenicol, and rifampicin. Derived from an MRSA epidemic.
- *K. pneumoniae* 3978: resistant to amikacin, gentamicin, amoxicillin, clavulanic acid, ampicillin, sulbactam, cefazolin, cephalothin, cefuroxime, cefoxitin, ceftazidime, ceftriaxone, cefepime, ertapenem, imipenem, meropenem, piperacillin-tazobactam, nitrofurantoin, ciprofloxacin, norfloxacin, nalidixic acid, trimethoprim-sulfamethoxazole, colistin, polymyxin B, and tigecycline. Isolated from a burn wound at HU.
- *A. baumannii* 141: resistant to ampicillin, ceftazidime, imipenem, meropenem, trimethoprim-sulfamethoxazole, amikacin, ciprofloxacin, levofloxacin, and gentamicin. Isolated from urine at HU.
- *P. aeruginosa* 3167: resistant to cefepime, ciprofloxacin, meropenem, piperacillin-tazobactam, and tobramycin. Isolated from a burn wound at the Instituto Adolfo Lutz in Presidente Prudente, São Paulo State, Brazil.
- *E. cloacae* 9434: resistant to amikacin, gentamicin, cefuroxime, cefoxitin, ceftazidime, ceftriaxone, cefepime, ertapenem, ciprofloxacin, and tigecycline. Isolated from tracheal secretion at HU.
- *E. coli* 5616: resistant to cefoxitin, imipenem, meropenem, aztreonam, ciprofloxacin, gentamicin, nitrofurantoin, tetracycline, and fosfomycin. Isolated from urine at HU.

### 4.2. Drugs

The drugs SER (50 mg, Novartis, Hyderabad, Telegana, India) and PAR (20 mg, Novartis, Telegana, India) were used in this study. Both drugs were crushed and diluted at a 1:9 ratio in a solution containing peptone (Himedia, Nashik, India), soy lectin (ACS Científica, Sumaré, Brazil), Tween 80 (Sigma-Aldrich, Steinheim, Germany), sodium chloride (Biotec, São José dos Pinhais, Brazil), monopotassium phosphate (Dinâmica, Indaiatuba, Brazil) and sodium phosphate (Dinâmica, Indaiatuba, Brazil) to neutralize the action of the preservatives within the medications.

### 4.3. Determination of Minimal Inhibitory Concentration (MIC)

The MIC was determined by the broth microdilution method, following the document M07-A10, published by the Clinical and Laboratory Standards Institute [49], adapted. Briefly, a 96-well plate was filled with Mueller-Hinton broth (MHB) (Himedia, Nashik, India) and subsequently treated with serial dilutions of SER or PAR, with concentrations ranging from 3.9 to 8125 µg/mL for SER, and 1 to 2040 µg/mL for PAR. Finally, each well received a bacterial inoculum to achieve a final concentration of 5 × 10^5^ CFU/mL in each well, and the 96-well plate was incubated at 37 °C overnight. The MIC was defined as the lowest concentration at which the tested antimicrobial showed no visible growth. The drug diluent, MHB containing only SER, and MH broth containing only PAR were used as sterility controls; bacteria inoculated in MHB were used as the viability and cell-growth controls. The test was performed in triplicate in 96-well plates on three separate occasions.

### 4.4. Determination of the Minimal Bactericidal Concentration (MBC)

The MBC was determined following the document M26-A, published by the National Committee for Clinical Laboratory Standards [50], with minor modifications. Briefly, the MBC was determined by taking aliquots from the 96-well plate used for the broth microdilution test. The aliquots from each well were diluted and plated on Mueller–Hinton agar in triplicates. After incubation for 24 h at 37 °C, bacterial colonies were counted to determine the CFU/mL. The MBC was defined as the lowest concentration capable of killing at least 99.9% of bacteria (a reduction of ≥3 log_10_ CFU/mL) after 24 h of antimicrobial treatment. The test was performed in triplicate in 96-well plates on three separate occasions.

### 4.5. Time–Kill Assay

The time–kill curve for the tested drugs was performed following the adapted guidelines in document M26-A, published by the National Committee for Clinical Laboratory Standards [50]. Briefly, tubes containing MHB were treated with antimicrobials at their respective MBCs and inoculated with bacteria to achieve a final concentration of 5 × 10^5^ CFU/mL. The tubes were incubated at 37 °C. Then, 100 µL aliquots were collected at 0, 2, 4, 7, 10, and 24 h time points, diluted, and plated onto MH agar in triplicate. The plates were incubated for 24 h at 37 °C. After this period, colony counts were performed on each plate to determine the bacterial concentration in each aliquot. The test was performed in triplicate on three separate occasions.

### 4.6. Electron Scanning Microscopy

The cells of *S. aureus* ATCC 25923 were gently washed and subjected to centrifugation (Universal 320 R, Hettich, Tuttlingen, Germany) (8000 rpm, 10 min, 25 °C) for pellet formation. The supernatants were discarded and the remaining pellets were resuspended in PBS containing SER and PAR at subinhibitory concentrations, and phosphate-buffered saline (PBS) was used as the control. Then, the suspensions were incubated for 3 h at 37 °C and 120 rpm, and washed two more times to remove residues. Once the residues were removed, 50 µL of each suspension and 50 µL of fixation buffer (0.1 M sodium cacodylate buffer, pH 7.2, containing 2.5% glutaraldehyde) were carefully spread out on poly-L-lysine-coated glass slides. Afterwards, the glass slides were submerged in fixation buffer for 20 h at 4 °C, followed by three 10 min washes in the same buffer, post-fixation with OsO4 for 1 h at room temperature, and another three 10 min washes. To dehydrate the cells fixed on the glass slides, sequential washes in ethanol 30, 50, 70 and 90%, as well as three 10 min washes in ethanol 100%, were performed, followed by critical point drying with CO_2_. Finally, the fixed bacterial cells were coated with gold and observed under a scanning electron microscope (Quanta 200, FEI Company, Hillsboro, OR, USA).

### 4.7. Determination of Subinhibitory Antibacterial Concentration for Mechanism Assays

To determine the subinhibitory concentrations of SER and PAR for antibacterial mechanism assays, a 4 h time–kill assay was performed. Suspensions of *Escherichia coli* ATCC 25922 and *Staphylococcus aureus* ATCC 25923, at approximately 1.5 × 10^8^ CFU/mL, were treated with SER and PAR at their MIC and ½ MIC in PBS, and incubated at 37 °C, under agitation (120 rpm). Then, 100 µL aliquots were collected at intervals of 0.5, 1, 2, 3, and 4 h, diluted, and plated onto MH agar in triplicate. The plates were incubated for 24 h at 37 °C. After this period, colony counts were performed to determine the bacterial concentration in each aliquot. The test was performed in triplicate on three separate occasions.

### 4.8. Membrane Permeability Evaluation

#### 4.8.1. Measurement of Permeability Using High-Molecular-Weight Dyes

Changes in membrane permeability were evaluated using the crystal violet assay [51,52], with minor modifications. Bacterial cultures were grown in MHB overnight. The bacterial cell suspension, containing approximately 1.5 × 10^8^ CFU/mL, was washed with PBS and subjected to centrifugation (8000 rpm, 10 min, 25 °C). The pellet containing the collected bacterial cells was resuspended in PBS containing SER and PAR at subinhibitory concentrations and incubated for 3 h at 37 °C and 120 rpm. PBS alone was used as a negative control, whereas Tween 80 and Triton X-100 at subinhibitory concentrations were used as positive controls. After another centrifugation (8000 rpm, 10 min, 25 °C), the cells were resuspended in PBS containing 0.001% (*m*/*v*) Evans blue (Merck, Darmstadt, Germany) and Rose Bengal (Inlab, São Paulo, Brazil) followed by incubation for 10 min. A final centrifugation (8000 rpm, 10 min, 25 °C) was performed, followed by the collection of the supernatants. The absorbance of the supernatants was quantified at 530 nm for Rose Bengal and 605 nm for Evans blue (LMR-96i microplate reader, locus, Cotia, Brazil). Solutions of these dyes at 0.001% (*m*/*v*) were used as controls to represent 100% absorption. The percentage of dye absorption by bacterial cells was determined using the following equation:

$$\mathrm{Dye}\;\mathrm{absorbed}\;\mathrm{by}\;\mathrm{bacteria}=100-\left(\frac{\mathrm{Supernatant}\;\mathrm{absorbance}\;\times 100}{\mathrm{Dye}\;\mathrm{solution}\;\mathrm{absorbance}}\right)$$

The test was performed in quadruplicate on four separate occasions.

#### 4.8.2. Quantification of Biomolecule Leakage

*E. coli* ATCC 25922 and *S. aureus* ATCC 25923 were cultured in MHB and incubated overnight. Aliquots of 1 mL from the bacterial cultures were centrifuged at 8000 rpm for 10 min, and the supernatants were discarded. The resulting pellets were resuspended in PBS to remove residual culture medium, and the suspensions were centrifuged. Again, the supernatants were discarded, and the pellets were resuspended in PBS treated with subinhibitory concentrations of SER and PAR for the test groups; Tween 80 and Triton X-100 were used as positive controls for cell wall damage. A negative control group was also prepared, resuspended only in PBS. The bacterial suspensions were incubated at 37 °C and 120 rpm for 3 h. After incubation, another centrifugation at 8000 rpm for 10 min was performed, followed by collection of the supernatants. The supernatants were analyzed using NanoDrop™ (Thermo Fisher Scientific, Waltham, MA, USA) to quantify total proteins at 280 nm and dsDNA at 260 nm, using the 260/280 nm ratio to assess the quality of the obtained genomic DNA. The assay was performed in triplicate on four separate occasions.

### 4.9. Efflux Pump Inhibition Evaluation

#### 4.9.1. Resistance Modulation Assay

The inhibitory activity of efflux pumps was determined using a modulation assay, with modifications [53,54]. The MIC of tetracycline (Sigma-Aldrich, Steinheim, Germany) was determined in the presence of SER and PAR at subinhibitory concentrations. *E. coli* XL1-Blue, resistant to TET owing to efflux pump activity, and *E. coli* K12, sensitive to TET, were used as test strains. MHB supplemented with tetracycline was used as the control; sterility and growth controls were also included. If SER and PAR modulate tetracycline activity by reducing its MIC against *E. coli* XL1-Blue, it is assumed that the tested SSRIs act as EPIs. The test was performed in triplicate on three separate occasions.

#### 4.9.2. Ethidium Bromide Accumulation Assay

The efflux of ethidium bromide was evaluated using an ethidium bromide accumulation and efflux assay [55,56,57]. Cultures of *E. coli* ATCC 25922 and *S. aureus* ATCC 25923 were grown in MHB overnight. The cells were centrifuged at 8000 rpm for 10 min, the supernatant was discarded, and the pellets were resuspended in 20 mM potassium phosphate buffer supplemented with 1 mM MgCl_2_ to remove residual culture medium. After another centrifugation, the pellets were resuspended in PBS supplemented with subinhibitory concentrations of SER or PAR, and incubated for 2 h at 37 °C and 120 rpm.

Ethidium bromide was then added to the suspensions at a final concentration of 5 µg/mL, and the suspensions were incubated for an additional hour at 37 °C and 120 rpm. An untreated suspension was used as the control. After incubation, the suspensions were centrifuged again, and the pellets were resuspended in 20 mM potassium phosphate buffer supplemented with 1 mM MgCl_2_ and 5% glucose. Subsequently, 100 µL of each suspension was transferred to a black-sided 96-well microplate. The fluorescence of the suspensions was quantified (GloMax Discover microplate reader, Promega, Madison, WI, USA, EUA) at excitation and emission wavelengths of 520 nm and 600 nm, respectively. The test was performed in quintuplicate on three separate occasions.

### 4.10. Oxidative Stress Evaluation

#### 4.10.1. Detection of the Effect of Antioxidants on Antibacterial Activity

The impacts of ascorbic acid (Dinâmica, Indaiatuba, Brazil) and glutathione (Sigma-Aldrich, Steinheim, Germany) antioxidants on the antimicrobial activity of SER and PAR were evaluated [40]. The MIC values of SER and PAR were determined in the presence of ascorbic acid and glutathione at subinhibitory concentrations. If glutathione and ascorbic acid antioxidants antagonize the antibacterial activity of SER and PAR, increasing their MICs, it is inferred that the antibacterial effect involves oxidative stress. AgNO_3_ (Sigma-Aldrich, Steinheim, Germany) and H_2_O_2_ (Biotec, São José dos Pinhais, Brazil) were used as positive controls for ROS production, and ampicillin was used as a negative control. The test was performed in triplicate on three separate occasions.

#### 4.10.2. Measurement of Reactive Oxygen Species

To measure the ROS generated by SER and PAR, the fluorescent indicator 2′,7′-dichlorofluorescein diacetate (H_2_DCFDA) was utilized [44]. Initially, bacterial cultures of *E. coli* ATCC 25922 and *S. aureus* ATCC 25923 were grown overnight in MHB. Then, the bacterial cultures were centrifuged at 8000 rpm for 10 min, washed with PBS to remove residual culture medium, and centrifuged again. The resultant supernatant was then resuspended in a solution of H_2_DCFDA at a final concentration of 50 µM, and incubated for 45 min at 37 °C in the dark. After the incubation period, the labeled bacterial suspensions were washed twice with PBS, and 100 µL of the labeled cells were added to 100 µL of solutions containing SER and PAR at subinhibitory concentrations. PBS was used as the negative control, and H_2_O_2_ served as the positive control. Finally, the 200 µL of labeled and treated bacterial suspensions was transferred to a black 96-well microplate with a clear bottom. Fluorescence was read at an emission wavelength of 500–550 nm and an excitation wavelength of 475 nm at 30, 60, 90, and 120 min. The test was performed in quadruplicate. The excitation wavelength of sertraline is 280 nm, with an emission at 560 nm, while paroxetine has an excitation wavelength of 244 nm and an emission at 339 nm [58]. Since none of these wavelengths overlap with those used in the assay, the autofluorescence of SER and PAR was disregarded.

### 4.11. Resistance Inducement Assay

The inducement of resistance to SER and PAR was performed according to the method described by Kanafani et al. [59], with adaptations. The inducement was carried out using reference strains of *E. coli* ATCC 25922 and *S. aureus* ATCC 25923. Initially, MHB was supplemented with one-eighth of the MIC of SER or PAR, followed by passages every 24 h of incubation at 37 °C until robust growth was achieved. Once robust growth was observed, the next passage was performed in MHB supplemented with double the concentration of the previous treatment. This process was repeated until 30 passages were completed. Controls consisted of untreated bacteria that underwent 30 passages.

### 4.12. Statistical Analysis

The results were analyzed using the software GraphPad Prism 9. The collected data were subjected to the Shapiro–Wilk normality test and Bartlett’s test of homogeneity of variances. If the assumptions of normality and homogeneity were met, an analysis of variance (ANOVA) test was performed, followed by Dunnett’s multiple comparisons test. If the assumptions were not met, data transformation was performed. When transformation was not possible, the non-parametric Kruskal–Wallis test was performed, followed by Dunn’s multiple comparisons test. For MIC and MBC comparisons specifically, the Wilcoxon test was used to compare the MICs and MBCs of SER and PAR, and the Mann–Whitney test was used to compare MICs and MBCs of standard strains and clinical isolates.

## 5. Conclusions

Both SER and PAR were able to eliminate Gram-positive and Gram-negative strains from the ESKAPEE group, including carbapenem-resistant strains, enabling the determination of MIC and MBC values for all the tested strains. The drugs under study were capable of killing bacteria within 2 to 24 h when used at MBC concentrations. Furthermore, SER and PAR showed bacteriostatic activity at MIC concentrations and bactericidal activity at higher concentrations, displaying slightly better activity against Gram-positive bacteria. This study shows that the antibacterial mechanisms of action of SER and PAR involve changes in membrane permeability, damage to the cell membrane, the inhibition of efflux pumps, and the ability to generate oxidative stress.

In summary, our study demonstrated the antibacterial activity of SER and PAR against bacteria from the ESKAPEE group, and investigated some of their antibacterial mechanisms of action. Therefore, both SSRIs prove to be an interesting possibility for drug repositioning, with the potential for application in clinical and hospital settings, particularly in the face of a scenario in which the options for treating multidrug-resistant bacteria are becoming increasingly restricted.

## Figures and Tables

**Table 1:** Minimal inhibitory concentration (MIC) and minimal bactericidal concentration (MBC) of sertraline (SER) and paroxetine (PAR) against reference strains and multidrug-resistant strains from the ESKAPEE group.

Bacteria	Sertraline (µg/mL)		Paroxetine (µg/mL)	
	MIC	MBC	MIC	MBC
*Escherichia coli* ATCC 25922	15	30	127.5	255
*Staphylococcus aureus* ATCC 25923	15	30	127.5	255
*Enterococcus faecium* 99455	15	30	60	127.5
*Staphylococcus aureus* BEC9393	60	60	127.5	255
*Klebsiella pneumoniae* 3978	30	30	127.5	1020
*Acinetobacter baumannii* 141	15	30	255	500
*Pseudomonas aeruginosa* 3167	127.5	500	127.5	2040
*Enterobacter cloacae* 9434	60	500	127.5	2040
*Escherichia coli* 5616	15	60	127.5	255

**Table 2:** Growth of *Escherichia coli* ATCC 25922 and *Staphylococcus aureus* ATCC 25923 treated with sertraline (SER) and paroxetine (PAR) at subinhibitory concentrations. (*) *p*-value ≤ 0.05, (**) *p*-value ≤ 0.01, (***) *p*-value ≤ 0.001.

*Escherichia coli* ATCC 25922	½ MIC (log10 CFU/mL)			MIC (log10 CFU/mL)		
	Sertraline	Paroxetine	Control	Sertraline	Paroxetine	Control
30 min	8.77	8.75	8.72	8.67	8.60	8.62
1 h	8.82	8.94	8.82	8.61 **	8.53 **	8.80
2 h	8.64 *	8.85 ***	8.59	8.59	8.40 **	8.72
3 h	8.71	8.58 *	8.79	8.66 **	8.26 *	8.80
4 h	8.61	8.47 **	8.76	8.47 *	7.86 **	8.89
*Staphylococcus aureus* ATCC 25923	½ MIC (log10 CFU/mL)			MIC (log10 CFU/mL)		
	Sertraline	Paroxetine	Control	Sertraline	Paroxetine	Control
30 min	8.78 *	7.87 *	8.32	8.56	7.95 *	8.32
1 h	8.54 **	7.57 **	8.10	8.32	7.63 **	8.10
2 h	8.58 **	7.86 *	8.14	8.53 **	7.67 ***	8.14
3 h	8.76 *	7.58 ***	8.48	8.31	7.58 **	8.48
4 h	8.53	7.73 **	8.82	9.01	7.45 **	8.82

**Table 3:** Minimal inhibitory concentration of tetracycline alone, or in combination with sertraline or paroxetine, at subinhibitory concentrations, against *Escherichia coli* XL1-Blue (resistant to tetracycline due to efflux pumps) and *Escherichia coli* K12 (sensitive to tetracycline).

Bacteria	Treatment	Mean Inhibitory Concentration (µg/mL)	SD
*Escherichia coli* XL1-Blue	Tetracycline	312.50	0.00
	Tetracycline + Sertraline	130.12 *	36.78
	Tetracycline + Paroxetine	11.88 ****	4.07
*Escherichia coli* K12	Tetracycline	0.225	0.08
	Tetracycline + Sertraline	0.15	0.00
	Tetracycline + Paroxetine	0.30	0.00

(*) *p*-value ≤ 0.05, (****) *p*-value ≤ 0.0001. SD, standard deviation.

**Table 4:** Minimal inhibitory concentrations of sertraline and paroxetine alone or in combination with glutathione and ascorbic acid against *Escherichia coli* ATCC 25922 and *Staphylococcus aureus* ATCC 25923.

Treatment	Mean Inhibitory Concentration	
	*Escherichia coli* ATCC 25922	*Staphylococcus aureus* ATCC 25923
Sertraline	25.5 µg/mL	27.0 µg/mL
Sertraline + Glutathione	13.7 µg/mL	62.5 µg/mL
Sertraline + Ascorbic Acid	15.6 µg/mL	62.5 µg/mL
Paroxetine	127.5 µg/mL	127.5 µg/mL
Paroxetine + Glutathione	209.2 µg/mL	250.0 µg/mL
Paroxetine + Ascorbic Acid	416.7 µg/mL	250.0 µg/mL
AgNO_3_	62.5 μM	93.5 μM
AgNO_3_ + Glutathione	>1000 μM	>1000 μM
AgNO_3_ + Ascorbic Acid	0.128 mM	-
H_2_O_2_	0.7 mM	2 mM
H_2_O_2_ + Glutathione	1.5 mM	32 mM
H_2_O_2_ + Ascorbic Acid	1 mM	-
Ampicillin	4 µg/mL	0.25 µg/mL
Ampicillin + Glutathione	1 µg/mL	0.25 µg/mL
Ampicillin + Ascorbic Acid	1 µg/mL	-

## Data Availability

Data are contained within the article.
